# miR-20a suppresses Treg differentiation by targeting *Map3k9* in experimental autoimmune encephalomyelitis

**DOI:** 10.1186/s12967-021-02893-4

**Published:** 2021-05-26

**Authors:** Yishu Wang, Chong Xie, Yaying Song, Weiwei Xiang, Jing Peng, Lu Han, Jie Ding, Yangtai Guan

**Affiliations:** Department of Neurology, Renji Hospital, Shanghai Jiaotong University School of Medicine, 160 Pujian Road, Shanghai, 200127 China

**Keywords:** miR-20a, Map3k9, Regulatory T cell, Experimental autoimmune encephalomyelitis, Inflammatory demyelinating diseases of the central nervous system

## Abstract

**Background:**

Experimental autoimmune encephalomyelitis (EAE) is a model for inflammatory demyelinating diseases of the central nervous system (CNS), a group of autoimmune diseases characterized by inflammatory infiltration, demyelination, and axonal damage. miR-20a is dysregulated in patients with CNS inflammatory demyelinating diseases; however, the function of miR-20a remains unclear. In this study, we intended to explore the role of miR-20a in EAE.

**Methods:**

The expression of miR-20a was detected by quantitative real-time PCR (qRT-PCR) in EAE mice and patients with MOG antibody-associated demyelinating diseases. CD4^+^ T cells of EAE mice were sorted, stimulated, and polarized with miR-20a knockdown. Activation and differentiation of CD4^+^ T cells were analyzed by flow cytometry*.* The expression of target gene *Map3k9* was detected by qRT-PCR and western blot experiments. The binding of miR-20a to the 3’ UTR of *Map3k9* was tested by luciferase assays*.* The feasibility of miR-20a as a therapeutic target to alleviate the severity of EAE was explored by intravenous administration of miR-20a antagomirs to EAE mice.

**Results:**

miR-20a was upregulated in splenocytes and lymph node cells, CD4^+^ T cells, and spinal cords of EAE mice. Moreover, miR-20a knockdown did not influence the activation of antigen-specific CD4^+^ T cells but promoted their differentiation into Treg cells. *Map3k9* was predicted to be a target gene of miR-20a. The expressions of Map3k9 and miR-20a were negatively correlated, and miR-20a knockdown increased the expression of Map3k9. In addition, miR-20a binded to the 3’ UTR of *Map3k9*, and simultaneous knockdown of miR-20a and Map3k9 counteracted the enhanced differentiation of Tregs observed when miR-20a was knocked down alone. Furthermore, injection of miR-20a antagomirs to EAE mice reduced the severity of the disease and increased the proportion of Treg cells in peripheral immune organs.

**Conclusions:**

miR-20a suppresses the differentiation of antigen-specific CD4^+^ T cells into Tregs in EAE by decreasing the expression of Map3k9. miR-20a antagomirs alleviate EAE, suggesting a new therapy for EAE and CNS inflammatory demyelinating diseases.

**Supplementary Information:**

The online version contains supplementary material available at 10.1186/s12967-021-02893-4.

## Background

Inflammatory demyelinating diseases of the central nervous system (CNS) are a group of autoimmune diseases targeting oligodendrocytes or support cells (such as astrocytes); these diseases are characterized by inflammatory infiltration, demyelination, and axonal damage, and examples include multiple sclerosis (MS), neuromyelitis optica spectrum disorder (NMOSD), and myelin oligodendrocyte glycoprotein (MOG) antibody-associated demyelinating disease [1–4]. Millions of patients worldwide suffer various symptoms, including visual, motor, and sensory disturbances [5], while therapies for these diseases are still quite limited.

To date, the exact mechanism resulting in CNS inflammatory demyelinating diseases is still unclear, and both innate and adaptive immune cells have been reported to be involved [6–8]. Most studies on the pathogenesis of CNS inflammatory demyelinating diseases have been carried out on an experimental autoimmune encephalomyelitis (EAE) model. EAE used to be the most widely accepted model for MS [9, 10]; however, in recent years, a growing number of studies have demonstrated that the disease which EAE reproduces is MOG antibody-associated demyelinating disease [11, 12]. Although the mechanism for the development of EAE is uncertain, autoreactive CD4^+^ T cells are generally recognized as the main cells mediating myelin damage [13, 14]. In the EAE model, IFN-γ-producing T helper (Th)1 and IL-17A-producing Th17 cells are considered crucial effector cells [9, 10, 15]. They infiltrate into CNS lesions in both EAE mice and patients [9, 10], and transplanted autoreactive Th1 and Th17 cells can induce EAE symptoms in wild-type (WT) recipient mice. They activate astrocytes and microglia and secrete proinflammatory cytokines such as IFN-γ, IL-17A/F, and GM-CSF to cause tissue damage [5, 9, 10, 13, 14, 16–18]. In contrast, adoptive transfer of CD25^+^Foxp3^+^ regulatory T (Treg) cells is able to ameliorate EAE symptoms, whereas the depletion of Tregs worsens the disease [19], indicating the essential role of Tregs in the suppression of the autoimmune response and maintenance of immune tolerance.

MicroRNAs (miRNAs) are a class of non-coding RNA molecules that play a necessary role in cell differentiation, proliferation, development, and survival by binding to the complementary 3’ UTR of target mRNAs, resulting in mRNA translational inhibition or degradation [20, 21]. In addition, miRNAs are frequently transcribed together as polycistronic primary transcripts that are processed into multiple individual mature miRNAs that are coordinated in function [22]. The miR-17–92 cluster is a typical polycistronic miRNA gene encoding 6 miRNAs (miR-17, miR-18a, miR-19a, miR-20a, miR-19b-1, and miR-92a-1). Identified in 2005, miR-17–92 was initially distinguished as an ‘oncomir’ because of its oncogenic nature in hematopoietic malignancies, medulloblastomas, lung cancer, colon cancer, and pancreatic cancer [23, 24]. However, in recent years, the effects of miR-17–92 on CD4^+^ T cells have been gradually revealed. Overexpressing miR-17–92 in CD4^+^ T cells results in a higher percentage of cells in the S phase when stimulated with antigen [25] and a lower percentage of cells in the Sub-G0 phase when stimulated with anti-CD3 [26], indicating that miR-17–92 promotes proliferation and survival of activated CD4^+^ T cells. In addition, naïve CD4^+^ T cells deficient in miR-17–92 differentiate into fewer Th1 and Th17 cells but more induced Treg (iTreg) cells, suggesting the important role of miR-17–92 in the differentiation of CD4^+^ T cells [27, 28]. Furthermore, individual miRNAs of miR-17–92 are upregulated or downregulated in the peripheral blood of patients, and the expressions of the miRNAs revert to normal levels with medical treatment or during remission [29–34]. In general, accumulating evidence has demonstrated that miR-17–92 is an essential regulator of CD4^+^ T cells and that the expression of miR-17–92 is altered in patients with CNS inflammatory demyelinating diseases; however, the precise role of miR-17–92 in the development of diseases and whether miR-17–92 affects diseases by modulating CD4^+^ T cells remain unknown.

In this study, we focused on miR-20a, a member of the miR-17–92 cluster. We first detected the upregulation of miR-20a in EAE mice and patients with MOG antibody-associated demyelinating diseases. We then explored the role of miR-20a in the activation and differentiation of antigen-specific CD4^+^ T cells in EAE. In addition, we found that *Map3k9* (mitogen-activated protein kinase kinase kinase 9) is the target gene of miR-20a in CD4^+^ T cells of EAE mice, as confirmed by luciferase assays and functional validation. Furthermore, we tested the therapeutic potential of miR-20a antagomirs and found that miR-20a antagomirs alleviated EAE.

## Methods

### Mice

C57BL/6 mice were purchased from Lingchang Biotechnology Company (Shanghai, China). The animals were housed and fed in a specific pathogen-free animal facility at the Experimental Animal Center of Renji Hospital. 8 to 12-week-old female mice were used for all experiments. Experiments were performed in accordance with the guidelines for animal care and were approved by the Animal Ethics and Welfare Committee of Renji Hospital affiliated to Shanghai Jiaotong University School of Medicine (Shanghai, China).

### EAE induction and evaluation

For EAE induction, myelin oligodendrocyte glycoprotein residues 35–55 (MOG_35–55_) peptide (Met-Glu-Val-Gly-Trp-Tyr-Arg-Ser-Pro-Phe-Ser-Arg-Val-Val-His-Leu-Tyr-Arg-Asn-Gly-Lys, GL Biochem Ltd, Shanghai, China) dissolved in PBS was emulsified with complete Freund’s adjuvant (CFA), which is composed of incomplete Freund’s adjuvant and Mycobacterium tuberculosis *H37Ra* (BD Difco, MI, USA). The final concentrations of MOG_35-55_ peptide and Mycobacterium tuberculosis *H37Ra* in the MOG_35-55_/CFA emulsion were 1.5 mg/ml and 4 mg/ml, respectively. Each mouse was injected subcutaneously with 200 μl MOG_35–55_/CFA emulsion near bilateral inguinal lymph nodes. In addition, 200 ng pertussis toxin (PTX; Millipore, Billerica, MA, USA) was administered intraperitoneally on the day of immunization (Day 0) and again 2 days later (Day 2).

Clinical assessment of EAE was performed daily after disease induction according to the following scoring system: 0, no clinical symptoms; 0.5, limp tail; 1, paralyzed tail; 1.5, hindlimb weakness, uncoordinated movement; 2, hindlimb paresis; 2.5, paralysis of one hindlimb; 3, paralysis of one hindlimb with the other hindlimb weakness; 3.5, complete paralysis of both hindlimbs; 4, hindlimb paralysis with forelimb paresis; 4.5, forelimb and hindlimb paralysis; and 5, moribund state or death.

### RNA extraction, reverse transcription, and quantitative real-time PCR

Total RNA (including mRNA and small RNA) was extracted from cell pellets and spinal cord tissues using an RNeasy Mini Kit (QIAGEN, Hilden, Germany) according to the manufacturer’s instructions. Reverse transcription was performed using a Mir-X™ miRNA First Strand Synthesis Kit (Cat. 638315, Takara, Shiga, Japan) for miRNA and a PrimeScript™ RT reagent Kit (Cat. RR037A, Takara, Shiga, Japan) for mRNA. For relative quantitative real-time PCR (qRT-PCR), SYBR Advantage qPCR Premix (Cat. 639676, Takara, Shiga, Japan) and SYBR Green master mix (Cat. RR820A, Takara, Shiga, Japan) were used for the cDNA of miRNA and mRNA following the manufacturer’s instructions. The reactions were performed in a LightCycler 480 System (Roche, Basel, Switzerland), and U6 snRNA and β-actin were used as endogenous controls of miRNA and mRNA, respectively. The expression of the miRNAs and mRNAs normalized to the endogenous control were calculated using the 2^−ΔΔCT^ method and are presented as the fold change relative to the control group. The primer sequences applied in this study are listed in Additional file 1: Table S1.

### Isolation of CD4^+^ T cells

For isolation of CD4^+^ T cells from EAE and control mice, mice were sacrificed at the peak stage of disease after immunization, and the spleens and lymph nodes were harvested. The tissues were ground on a 40-μm strainer to prepare single-cell suspensions. After lysis of erythrocytes, splenocytes and lymph node cells were pelleted to isolate CD4^+^ T cells by magnetic bead cell sorting (MACS) according to the manufacturer’s instructions (Miltenyi Biotech, Bergisch Gladbach, Germany). In brief, the cell pellet was resuspended in MACS buffer and incubated with biotin-antibody cocktail for 5 min at 2–8 °C. After that, anti-biotin microbeads were added, for isolation of naïve CD4^+^ T cells, CD44 microbeads were also added. Then the mixture was incubated for 10 min at 2–8 °C. After incubation, the cells were separated with an LS Column in a MACS Separator, and the unlabeled cells in the flow-through, which are the CD4^+^ T cells, were collected.

### Transfection of antagomirs and small interfering RNAs

For miR-20a and Map3k9 silencing, a chemically modified ssRNA oligonucleotide, miR-20a antagomir, was applied to knock down the expression of miR-20a, and small interfering RNA (siRNA) duplexes of Map3k9 were used for the knockdown of Map3k9. The antagomirs, siRNAs, and negative control were synthesized by GenePharma Ltd. (Shanghai, China), and their sequences were as follows. miR-20a antagomir: 5’-CUACCUGCACUAUAAGCACUUUA-3’; Map3k9 siRNA: sense, 5’-GGACCAGCUAACGACUAUATT -3’, antisense, 5’- UAUAGUCGUUAGCUGGUCCTT -3’; NC antagomir: 5’- CAGUACUUUUGUGUAGUACAA -3’. The CD4^+^ T cells isolated from splenocytes and lymph node cells were transfected with the miR-20a antagomirs (200 nM), Map3k9 siRNA (400 nM), or negative control using Entranster™-R4000 (Cat. 4000-3, Engreen, Beijing, China) and harvested 12 h after transfection for the following experiments.

### Treg polarization of naïve or MOG-specific CD4^+^ T cells in vitro

Cells were cultured in complete RPMI medium (10% fetal bovine serum, 2 mM GlutaMAX™ Supplement (Thermo Fisher Scientific, Waltham, MA, USA), 25 mM HEPES, 55 μM 2-mercaptoethanol, mycoplasma prophylactic reagent, and penicillin/streptomycin) in 24-well plates (3 × 10^6^ cells/ml). To induce the differentiation of naïve CD4^+^ T cells into Treg cells, an anti-CD3e (145-2C11, 10 μg/ml, Thermo Fisher Scientific, Waltham, MA, USA) antibody and anti-CD28 antibody (37.51, 10 μg/ml, Thermo Fisher Scientific, Waltham, MA, USA) were used to stimulate the cells, and recombinant mouse IL-2 (20 ng/ml, R&D Systems, Minneapolis, MN, USA) and mouse TGF-β1 (20 ng/ml, PeproTech, Rocky Hill, NJ, USA) were added to the medium for Treg polarization. Naïve CD4^+^ T cells stimulated with anti-CD3 and anti-CD28 antibodies without IL-2 or TGF-β1 were cultured as a control. The cells were polarized for 4 days before harvest.

To polarize MOG-specific CD4^+^ T cells into Treg cells, CD4^+^ T cells isolated from EAE were mixed with the remaining splenocytes and lymph node cells after transfection. MOG_35–55_ (40 μg/ml, GL Biochem Ltd, Shanghai, China) was used to stimulate the cells in the culture medium, and recombinant mouse IL-2 (20 ng/ml, R&D Systems, Minneapolis, MN, USA) and mouse TGF-β1 (20 ng/ml, PeproTech, Rocky Hill, NJ, USA) were added for Treg polarization. The cells were polarized for 5 days before harvesting.

### Flow cytometry

To analyze the activation and polarization of CD4^+^ T cells, the cells were first cultured for 3 to 5 days with stimulation as mentioned above. In some situations, the cells were restimulated with a cell stimulation cocktail (plus protein transport inhibitors) (Thermo Fisher Scientific, Waltham, MA, USA) for 5 h before harvesting if intracellular staining for cytokines was needed. For flow staining, the cells were first surface stained with antibodies against CD4, CD69, CD62L, and CD25 conjugated to fluorochromes, and then the cells were fixed and permeabilized with the Foxp3/Transcription Factor Staining Buffer Set (Thermo Fisher Scientific, Waltham, MA, USA). Then, intracellular staining for Foxp3, IFN-γ, and IL-17A was performed, followed by fixation using 2% paraformaldehyde. Isotype plus fluorescence minus one (FMO) controls were used for gating and nonspecific staining exclusion. All antibodies used in this study were purchased from BD Biosciences. Data were acquired on a BD LSRFortessa X-20 or BD Accuri C6 Plus instrument and analyzed with FlowJo software.

### Western blot

Spinal cord tissues were lysed with RIPA lysis buffer (Beyotime, Shanghai, China) containing protease and phosphatase inhibitors (Beyotime, Shanghai, China) and 5 mM EDTA to extract total protein. Protein concentration was measured using a Pierce™ BCA Protein Assay Kit (Thermo Fisher Scientific, Waltham, MA, USA). Equal amounts of protein (20 μg) for each sample were separated by 8% SDS-PAGE and transferred onto polyvinylidene fluoride (PVDF) membranes. Nonspecific binding was blocked by 5% BSA for 2 h at room temperature. The membranes were then incubated with anti-MLK1 (Map3k9) antibody (1:1000, Cat. #5029, CST, Danvers, MA, USA) and anti-β-actin antibody (1:2000, Cat. WB0196, Weiao, Shanghai, China) overnight at 4 °C, followed by incubation with HRP-conjugated secondary antibodies for 2 h at room temperature. Blots were visualized using ECL substrate (Weiao, Shanghai, China) on a ChemiDoc™ imaging system (Bio‐Rad, Danvers, MA, USA).

### miR-20a knockdown using antagomirs in vivo

To knock down the expression of miR-20a, chemically modified single-stranded miR-20a antagomirs (purchased from GenePharma Ltd, Shanghai, China) were used. miR-20a antagomirs in the transfection complexes were delivered by tail vein injection to EAE mice. The transfection complexes were prepared with Entranster™-in vivo (Engreen, Beijing, China) according to the manufacturer’s instructions. The miR-20a antagomirs were administered intravenously for 3 days from the day that clinical symptoms appeared (onset stage). For each mouse, 100 μg miR-20a antagomirs were injected for the first two days, and 50 μg was injected for the third day.

### 3’ UTR luciferase assay

The wild-type 3’ UTR of *Map3k9* containing the predicted binding site, mutant 3' UTR, or NC 3' UTR was amplified by PCR and cloned into a luciferase reporter vector (PGL3-CMV-LUC-MCS; Genomeditech, Shanghai, China) by Genomeditech Co., Ltd. Each vector, along with a Renilla luciferase vector (pRL-TK) and miR-20a mimics or control NC mimics, was transfected into HEK-293 cells using HG transgene reagent (Genomeditech, Shanghai, China). Cells were harvested and lysed at 48 h post-transfection. The luciferase activity was measured by an Infinite M1000 plate reader (Tecan, Switzerland), and the results are shown as relative light units (RLUs). The RLU of luciferase reporter containing 3’ UTR/RLU of Renilla luciferase ratios were normalized to that of cells cotransfected with luciferase reporter vector containing NC 3’ UTR, NC mimics, and pRL-TK. The values of the normalized RLU ratios were used for statistical analysis.

### Histological analysis

The spinal cords harvested from EAE mice were first fixed with 4% paraformaldehyde, and then the lumbar enlargements were embedded in paraffin and cut into 5-μm-thick sections, followed by hematoxylin and eosin (H&E) and Luxol fast blue staining. The number of infiltrating inflammatory cells was counted in the H&E-stained sections to represent the severity of inflammation, while the demyelination severity is expressed as the percentage of demyelinated area to total white matter area in Luxol fast blue stained sections.

### Statistical analysis

Statistical analysis was carried out using GraphPad Prism software (Version 8.3.1). The Shapiro–Wilk test was used for normality analysis of the data distribution. The unpaired Student’s *t*-test or paired Student’s *t*-test was used for significant difference analysis between two groups. The correlation between the expression of miR-20a and Map3k9 was identified with simple linear regression analysis. To compare the severity of disease in the two groups of EAE mice over the entire period of the disease, the area under the curve (RUC) for each mouse was calculated, and the difference was identified with Student’s *t*-test. In addition, to compare the severity of disease on each individual day, the clinical scores of the EAE mice from the two groups were analyzed by the Mann–Whitney test. Data are expressed as the mean ± SEM, and a *p*-value < 0.05 was considered significant.

## Results

### miR-20a is increased in EAE mice and patients with MOG antibody-associated demyelinating diseases

**Fig. 1:**
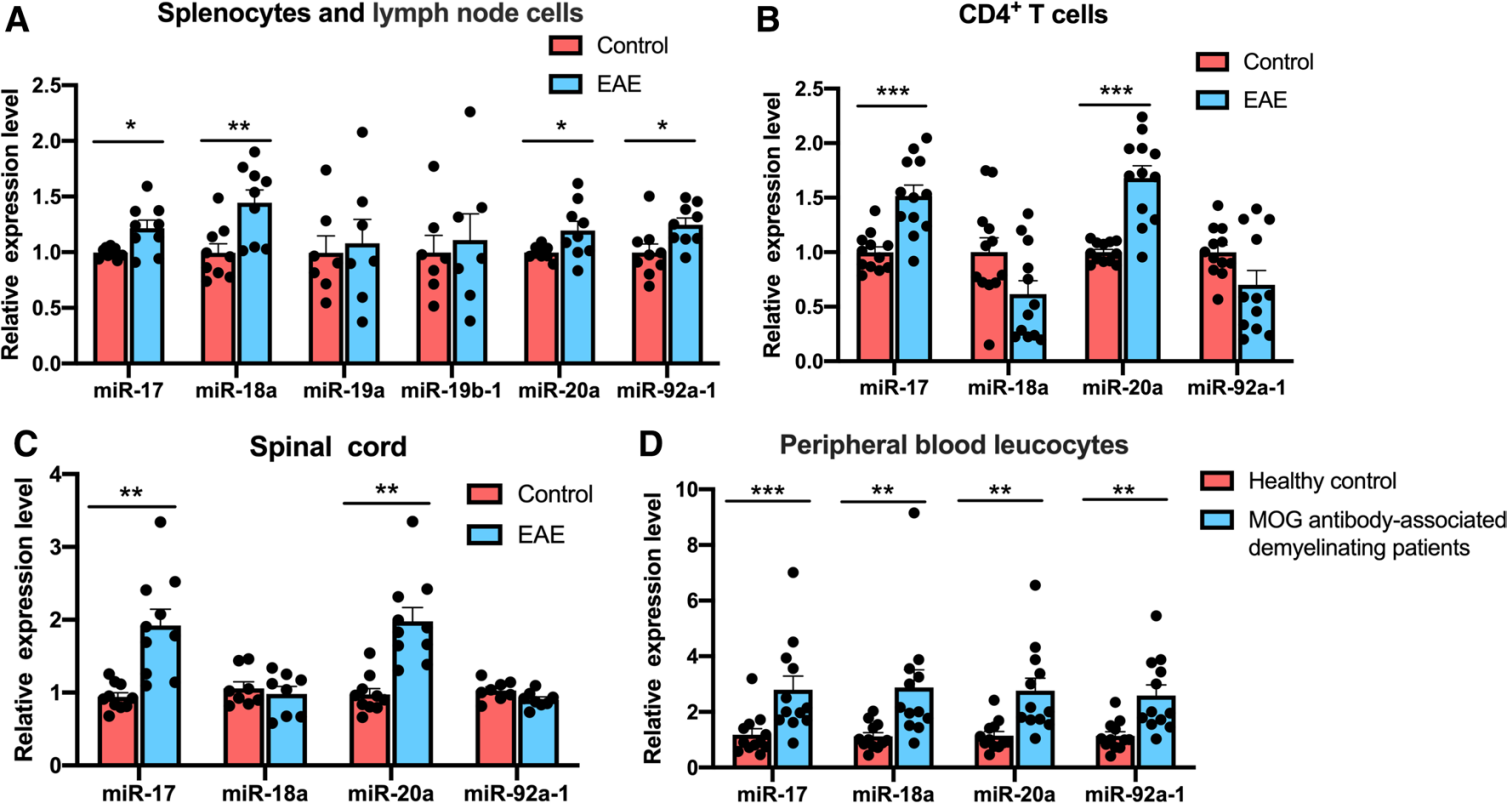
Different expression levels of miR-17–92 cluster in EAE mice and MOG antibody-associated demyelinating patients. **A** The expression levels of miR-17–92 cluster members in splenocytes and lymph node cells of EAE and control mice (n = 7–9). **B** The expression levels of miR-17–92 cluster members in isolated peripheral CD4^+^ T cells of EAE and control mice (n = 12). **C** The expression levels of miR-17–92 cluster members in spinal cord tissues of EAE and control mice (n = 8–10). **D** The expression levels of miR-17, miR-18a, miR-20a, and miR-92a-1 in the peripheral blood leucocytes of MOG antibody-associated demyelinating patients and healthy controls (HC) (n = 12). Data are shown as mean ± SEM. *p < 0.05, ** p < 0.01, *** p < 0.001 using unpaired Student’s *t*-test

To determine whether the miR-17-92 cluster is involved in the inflammatory response during EAE progression, we first tested the expression of the 6 miRNAs in the miR-17-92 cluster in splenocytes and lymph node cells. Compared to the control group, the expression levels of miR-17, miR-18a, miR-20a, and miR-92a-1 were increased in EAE mice, while miR-19a and miR-19b-1 were expressed equivalently between the two groups (Fig. 1A). We next detected the expression of miR-17, miR-18a, miR-20a, and miR-92a-1 in CD4^+^ T cells isolated from EAE and control mice, and miR-17 and miR-20a were found to be upregulated in EAE mice (Fig. 1B). Furthermore, the same results for miR-17 and miR-20a were also observed in spinal cord tissue of EAE and control mice (Fig. 1C). To determine the importance of the miRNAs in patients, we tested the expression level of the miRNAs in peripheral blood leukocytes from patients with MOG antibody-associated demyelinating diseases. Increased expression levels of miR-17, miR-18a, miR-20a, and miR-92a-1 were detected in peripheral blood leukocytes from patients compared with healthy controls (Fig. 1D), which were consistent with the results in splenocytes and lymph node cells from EAE mice. The above findings indicated that miR-17-92 cluster may be involved in inflammatory demyelinating diseases of the CNS. Moreover, miR-17 and miR-20a may play an important role in the development of EAE by affecting CD4^+^ T cells. The function of miR-17 in EAE mice has been previously reported [35], while the function of miR-20a remains unclear. We, therefore, chose miR-20a for further study.

### miR-20a does not affect CD4^+^ T cell activation in EAE

**Fig. 2:**
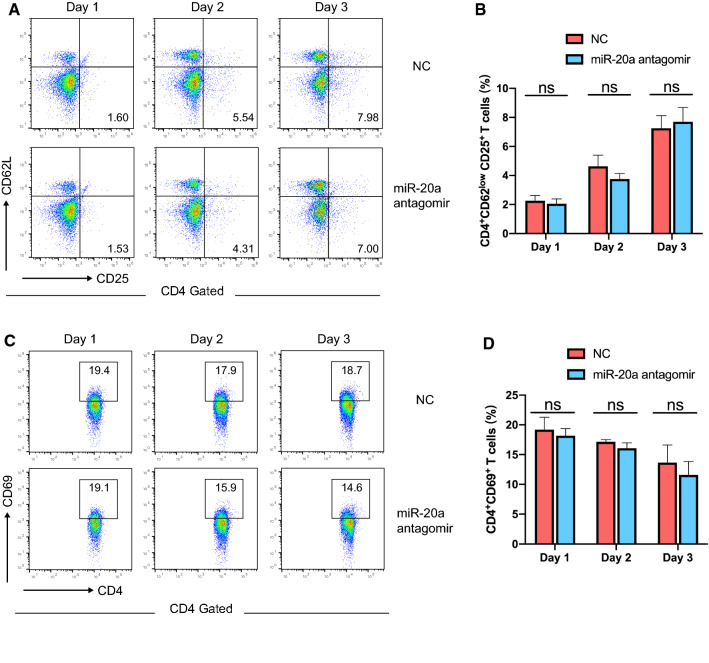
miR-20a does not affect the activation of MOG-specific CD4^+^ T cells in EAE mice. **A**, **B** The percentages of CD62L^low^CD25^+^ cells in the CD4^+^ gate, as measured by flow cytometry on Day 1, Day 2 and Day 3, separately. **C**, **D** The percentages of CD69^+^ cells in the CD4^+^ gate, as measured by flow cytometry on Day 1, Day 2 and Day 3, separately. The flow cytometry plots shown are representative of 3 independent experiments. Data are presented as mean ± SEM and ns represents no significant difference

We first explored whether miR-20a affects the activation of MOG-specific CD4^+^ T cells in EAE mice. To knock down the expression level of miR-20a, chemically modified antagomirs that are complementary to mature miR-20a were used. The knockdown efficiency of the miR-20a antagomirs is shown in Additional file 2: Figure S1. Isolated CD4^+^ T cells from the spleens and lymph nodes of EAE mice were transfected with miR-20a or negative control (NC) antagomirs and stimulated with MOG_35-55_ peptide for 3 days. Every day, activated CD4^+^ T cells defined as CD4^+^CD62L^low^CD25^+^ or CD4^+^CD69^+^ were analyzed by flow cytometry. The results showed that neither the percentage of CD4^+^CD62L^low^CD25^+^ (Fig. 2A, B) nor that of CD4^+^CD69^+^ T cells (Fig. 2C, D) was significantly different between the NC and miR-20a antagomir groups. The data indicated that miR-20a does not affect the activation of MOG-specific CD4^+^ T cells in EAE.

### miR-20a suppresses Treg differentiation in EAE

**Fig. 3:**
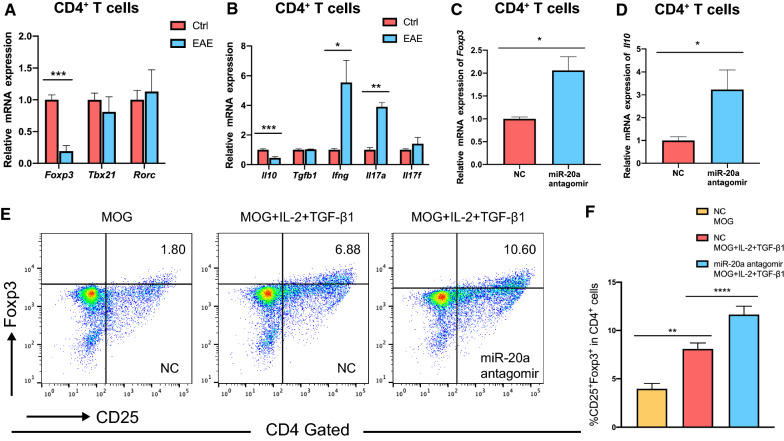
miR-20a suppresses Treg differentiation in EAE. **A** The mRNA levels of characteristic transcription factors of Treg (*Foxp3*), Th1 (*Tbx21*) and Th17 (*Rorc*) cells in CD4^+^ T cells of EAE and control mice. **B** The mRNA levels of characteristic cytokines of Treg (*Il-10*, *Tgfb1*), Th1 (*Ifng*) and Th17 (*Il-17a*, *Il-17f*) cells in CD4^+^ T cells of EAE and control mice. **C** The mRNA levels of *Foxp3* in Treg-polarized CD4^+^ T cells with miR-20a or NC antagomirs transfected. **D** The mRNA levels of *Il-10* in Treg-polarized CD4^+^ T cells with miR-20a or NC antagomirs transfected. **E**, **F**. The percentages of CD25^+^Foxp3^+^ cells in CD4^+^ T cells of EAE mice after a 5-day MOG_35-55_ stimulation and Treg polarization with miR-20a or NC antagomirs transfected. The flow cytometry plots shown are representative of 6 independent experiments. Data are shown as mean ± SEM. * p < 0.05, ** p < 0.01, **** p < 0.0001 using unpaired (**A**–**D**) and paired (**F**) Student’s *t*-test

Since miR-20a does not affect the activation of CD4^+^ T cells in EAE, we next hypothesized that miR-20a is involved in the differentiation of CD4^+^ T cells. Th1 and Th17 cells are crucial effector cells in EAE initiation and progression [36], while Treg cells suppress excessive autoimmune responses [37]. We first examined the mRNA levels of characteristic cytokines and transcription factors of Treg, Th1, and Th17 cells in CD4^+^ T cells of EAE and control mice. The mRNA expression of Foxp3 was reduced in EAE mice compared to the control group (Fig. 3A). Similarly, IL-10, the major anti-inflammatory cytokine secreted by Tregs, was also downregulated in EAE mice, though the expression of TGF-β1, another cytokine produced by Tregs, was comparable between the two groups (Fig. 3B). It should be noted that although IFN-γ and IL-17A were upregulated in EAE mice, the mRNA expression levels of T-bet (*Tbx21*) and RORγt (*Rorc*) were equivalent across the two groups (Fig. 3A, B). Thus, we speculated that miR-20a may affect the differentiation of Treg cells. Naïve CD4^+^ T cells (CD4^+^CD25^−^CD44^−^) isolated from naïve mice were Treg-polarized in vitro for 4 days, and we found that the mRNA levels of both Foxp3 (Fig. 3C) and IL-10 (Fig. 3D) significantly increased in CD4^+^ T cells with miR-20a knockdown. We next polarized CD4^+^ T cells from EAE mice into Tregs with IL-2 and TGF-β1 under the MOG_35-55_ stimulation. It was found that IL-2 and TGF-β1 successfully induced the differentiation of MOG-specific CD4^+^ T cells into Tregs under the MOG_35-55_ stimulation (Fig. 3E, F). Furthermore, the Treg differentiation was enhanced in the miR-20a knockdown group compared to the NC group. (Fig. 3E, F). The results collectively indicated that miR-20a suppresses the differentiation of Treg cells while reducing the expression level of miR-20a promotes Treg differentiation.

### *Map3k9* is a potential target gene of miR-20a

**Fig. 4:**
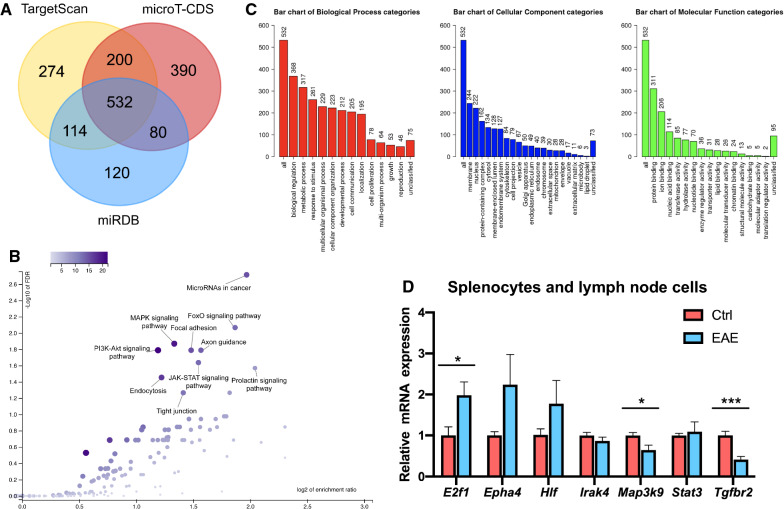
Prediction and initial validation of the target genes of miR-20a. **A** The potential target genes of miR-20a predicted by TargetScan, microT-CDS, and miRDB. 532 overlapping targets predicted by the three online programs were then functional enrichment analyzed. **B** a GO slim summary for the 532 target genes annotated by the online WebGestalt tool. **C** Functional annotation of the 532 overlapping genes based on KEGG pathway Database by the online WebGestalt tool. **D** The mRNA expression levels of 7 selected potential target genes in EAE and control mice (n = 6–9). Data are shown as mean ± SEM. * p < 0.05, *** p < 0.001 using unpaired Student’s *t*-test

We next investigated the mechanism by which miR-20a regulates the differentiation of Treg cells. We identified potential downstream gene targets of miR-20a using three online prediction tools based on the binding sites in the 3’ UTR, including TargetScan (www.targetscan.org), microT-CDS (http://www.microrna.gr/webServer) and miRDB (www.mirdb.org) (Fig. 4A). Among all of the putative gene targets, 532 overlapping targets of the three programs were functional enrichment analyzed on GO and KEGG pathway databases by the WebGestalt online tool (http://www.webgestalt.org) (Fig. 4B, C). We selected some potential gene targets that are functionally involved in inflammatory responses and tested their mRNA levels in splenocytes and lymph node cells of EAE and control mice. The expression of Map3k9 (mitogen-activated protein kinase kinase kinase 9) was downregulated in EAE mice (Fig. 4D), which makes *Map3k9* a potential gene target of miR-20a. Tgfbr2 (transforming growth factor beta receptor 2) was also downregulated in EAE mice; however, we next found that Tgfbr2 was upregulated in CD4^+^ T cells of EAE mice, which refuted the speculation that *Tgfbr2* was a miR-20a target gene (data not shown).

### Expression of Map3k9 is influenced by miR-20a

**Fig. 5:**
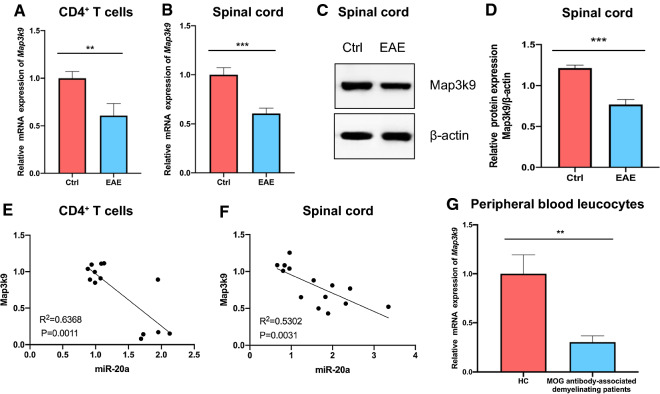
The expression of Map3k9 is influenced by miR-20a. **A** The mRNA expression level of Map3k9 in CD4^+^ T cells from EAE and control mice. **B** The mRNA expression level of Map3k9 in spinal cord tissues from EAE and control mice. **C**, **D**. The protein expression level of Map3k9 in spinal cord tissues from EAE and control mice tested by western blot. **E** Linear regression analysis of the correlation between the expression of miR-20a and Map3k9 in CD4^+^ T cells from EAE and control mice. **F** Linear regression analysis of the correlation between the expression of miR-20a and Map3k9 in spinal cord tissues from EAE and control mice. **G** The mRNA level of Map3k9 in the peripheral blood leucocytes of MOG antibody-associated demyelinating patients and HCs. Data are shown as mean ± SEM. ** p < 0.01, *** p < 0.001 using unpaired Student’s *t*-test

Since Map3k9 was downregulated in EAE mice when miR-20a was upregulated, we next investigated the correlation between the expression of Map3k9 and miR-20a. The mRNA level of Map3k9 was decreased in CD4^+^ T cells in EAE mice (Fig. 5A). Furthermore, the mRNA and protein levels of Map3k9 were also decreased in the spinal cords of EAE mice (Fig. 5B–D). In addition, by linear regression analysis, a significant negative correlation between the mRNA levels of miR-20a and Map3k9 was shown in both CD4^+^ T cells and spinal cord tissues from EAE and control mice (Fig. 5E, F). We finally validated this result in patients and found that the mRNA level of Map3k9 was decreased in peripheral blood leukocytes from patients with MOG antibody-associated demyelinating disease compared to healthy controls (Fig. 5G). Taken together, these results indicate that the expression of Map3k9 is influenced by that of miR-20a.

### *Map3k9* is a target gene of miR-20a to suppress Treg differentiation in EAE

**Fig. 6:**
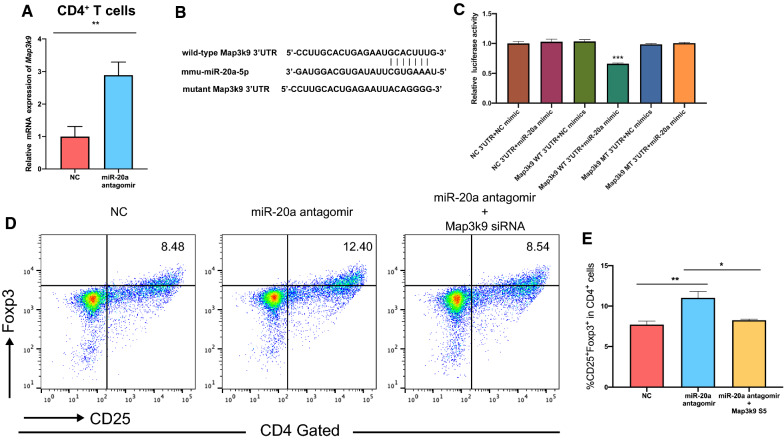
Map3k9 is the target gene of miR-20a to suppress Treg differentiation in EAE. **A** The expression of Map3k9 was upregulated in CD4^+^ T cells with miR-20a knockdown. **B** Sequences of WT and mutant 3’ UTR of *Map3k9* and miR-20a mimics used in the luciferase assays. **C** Relative luciferase activity of the reporter carrying the WT or mutant *Map3k9* 3’ UTR or NC 3’ UTR co-transfected with miR-20a mimics or NC mimics into HEK-293 cells. The activity of Renilla luciferase was used to eliminate variances between groups. **D**, **E** Flow cytometric analysis of the CD4^+^ T cells transfected with NC or miR-20a antagomirs, or miR-20a antagomirs + Map3k9 siRNA after stimulated with MOG_35-55_ peptide in Treg-polarizing condition for 5 days. The percentages of CD25^+^Foxp3^+^ cells are shown in the CD4^+^ gate. The flow cytometry plots shown are representative of 4 independent experiments. Data are shown as mean ± SEM. * p < 0.05, ** p < 0.01, *** p < 0.001 using unpaired (**A**, **C**) and paired (**E**) Student’s *t*-test

To determine whether *Map3k9* is the target gene for miR-20a to suppress Treg differentiation in EAE, we first knocked down the expression of miR-20a in CD4^+^ T cells and observed a significantly increased expression of Map3k9 (Fig. 6A). According to the results of target gene prediction, a putative binding site for miR-20a in the 3’ untranslated region (UTR) of *Map3k9* was identified. We next performed luciferase reporter assays to detect the binding of miR-20a and the 3’ UTR of *Map3k9* (Fig. 6B). The results showed that miR-20a specifically suppressed the luciferase activity of the reporter containing the wild-type 3’ UTR of *Map3k9* while the reporter containing a mutant or NC 3’ UTR was not affected, indicating effective and specific binding of miR-20a to the predicted binding site in *Map3k9* (Fig. 6C). At this point, we have demonstrated that *Map3k9* is a target gene of miR-20a.

We finally explored whether miR-20a inhibits the differentiation of Tregs in EAE mice by reducing the expression of Map3k9. To this end, isolated CD4^+^ T cells were transfected with antagomirs or were cotransfected with miR-20a antagomirs and Map3k9 siRNA (The knockdown efficiency of the siRNA is shown in Additional file 3: Figure S2). The cells were polarized into Tregs under MOG_35-55_ stimulation as mentioned above. The results showed that CD4^+^ T cells transfected with the miR-20a antagomirs alone displayed an enhanced capacity to differentiate into Treg cells, which was consistent with the previous results (Fig. 6D, E). However, the results did not show any difference in Treg differentiation between the CD4^+^ T cells transfected with miR-20a antagomirs + Map3k9 siRNA and the NC antagomirs (Fig. 6D, E). The findings suggested that miR-20a suppresses differentiation of MOG-specific CD4^+^ T cells into Tregs in EAE mice by reducing the expression of Map3k9.

Collectively, our results suggest that *Map3k9* is a functional target of miR-20a to suppress Treg differentiation in EAE.

### miR-20a antagomir treatment alleviates the severity of EAE in vivo

**Fig. 7:**
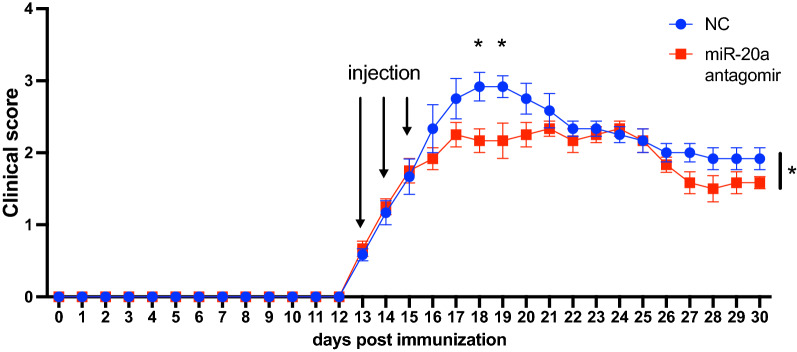
miR-20a antagomir treatment alleviates the clinical symptoms of EAE mice. The EAE mice received consecutive intravenous injections of antagomirs at the onset of the disease for 3 days and the observation period was 30 days post immunization (n = 6 per group). Data are shown as mean ± SEM. * p < 0.05 using unpaired Student’s *t*-test for comparing the area under curve (AUC) and Mann–Whitney test for comparing clinical scores for individual days

As described above, we demonstrated that miR-20a suppresses Treg differentiation of MOG-specific CD4^+^ T cells in EAE mice. We next explored whether miR-20a knockdown in EAE mice could alleviate the severity of EAE in vivo. We injected EAE mice intravenously with miR-20a or the NC antagomirs for 3 consecutive days at the onset of the disease and observed the clinical scores until the remission stage. The highest clinical score for mice injected with miR-20a antagomirs was 2.5 compared to 3.5 in mice injected with the NC antagomirs. In addition, as shown in Fig. 7, the average area under the scoring curve of the miR-20a antagomir group throughout the observation period was smaller than that of the NC group. For the individual days, the scores for the miR-20a antagomir group on Day 18 and Day 19 postimmunization were lower than those for the NC group. All of the above results indicated that miR-20a knockdown with antagomirs attenuates the clinical symptoms of EAE.

**Fig. 8:**
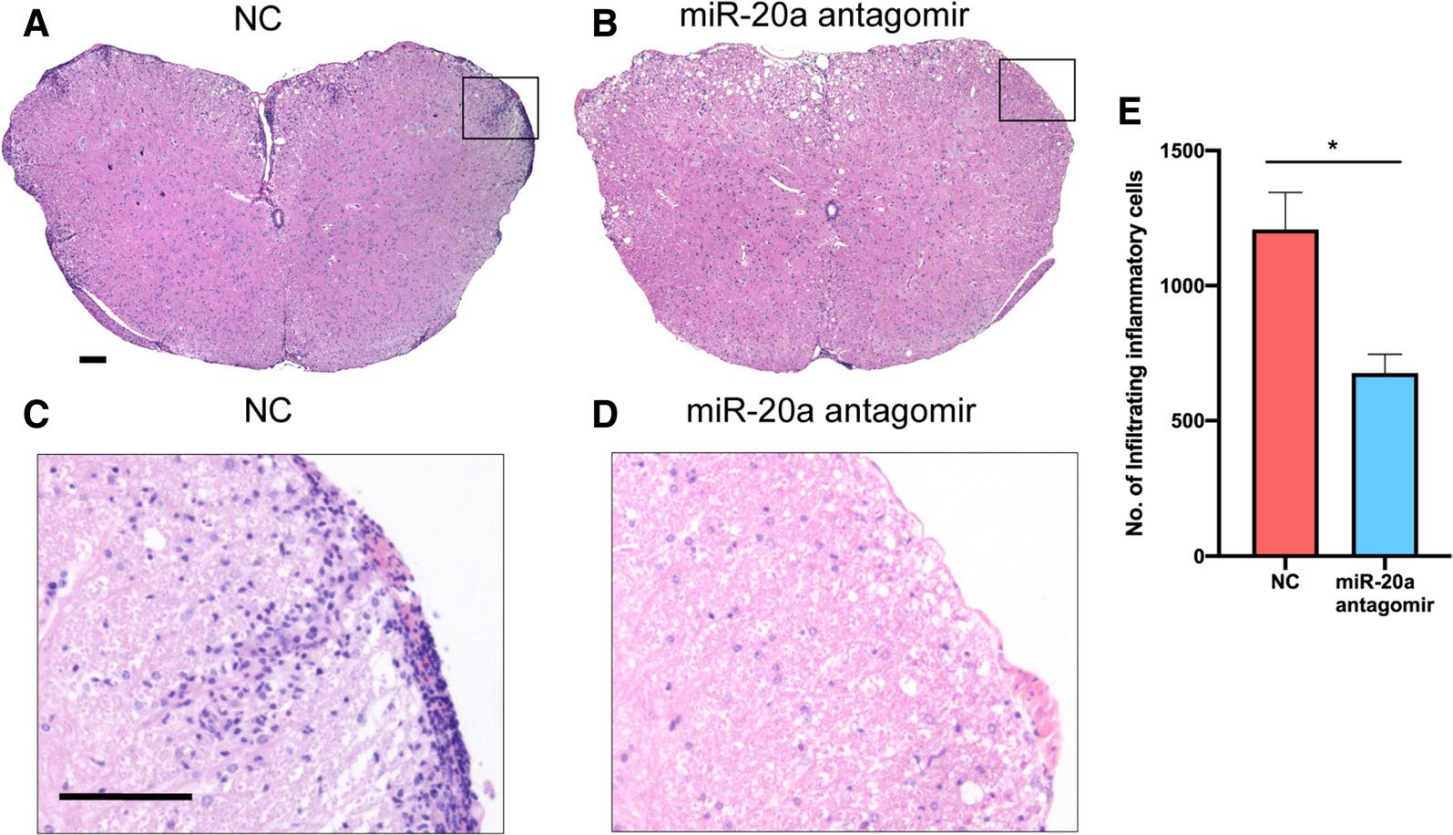
miR-20a antagomir treatment reduces the infiltrating inflammatory cells in spinal cords of EAE mice. **A** Representative H&E staining of complete spinal cord sections from NC group (taken at × 10 magnification). **B** Representative H&E staining of complete spinal cord sections from miR-20a antagomir group (taken at × 10 magnification). **C** Representative H&E staining of partial spinal cord sections from NC group (taken at × 20 magnification). **D** Representative H&E staining of partial spinal cord sections from miR-20a antagomir group (taken at × 20 magnification). **E** Quantification of the infiltrating inflammatory cells in the two groups. Scale bars correspond to 100 μm. Data are shown as mean ± SEM. * p < 0.05 using unpaired Student’s *t*-test

**Fig. 9:**
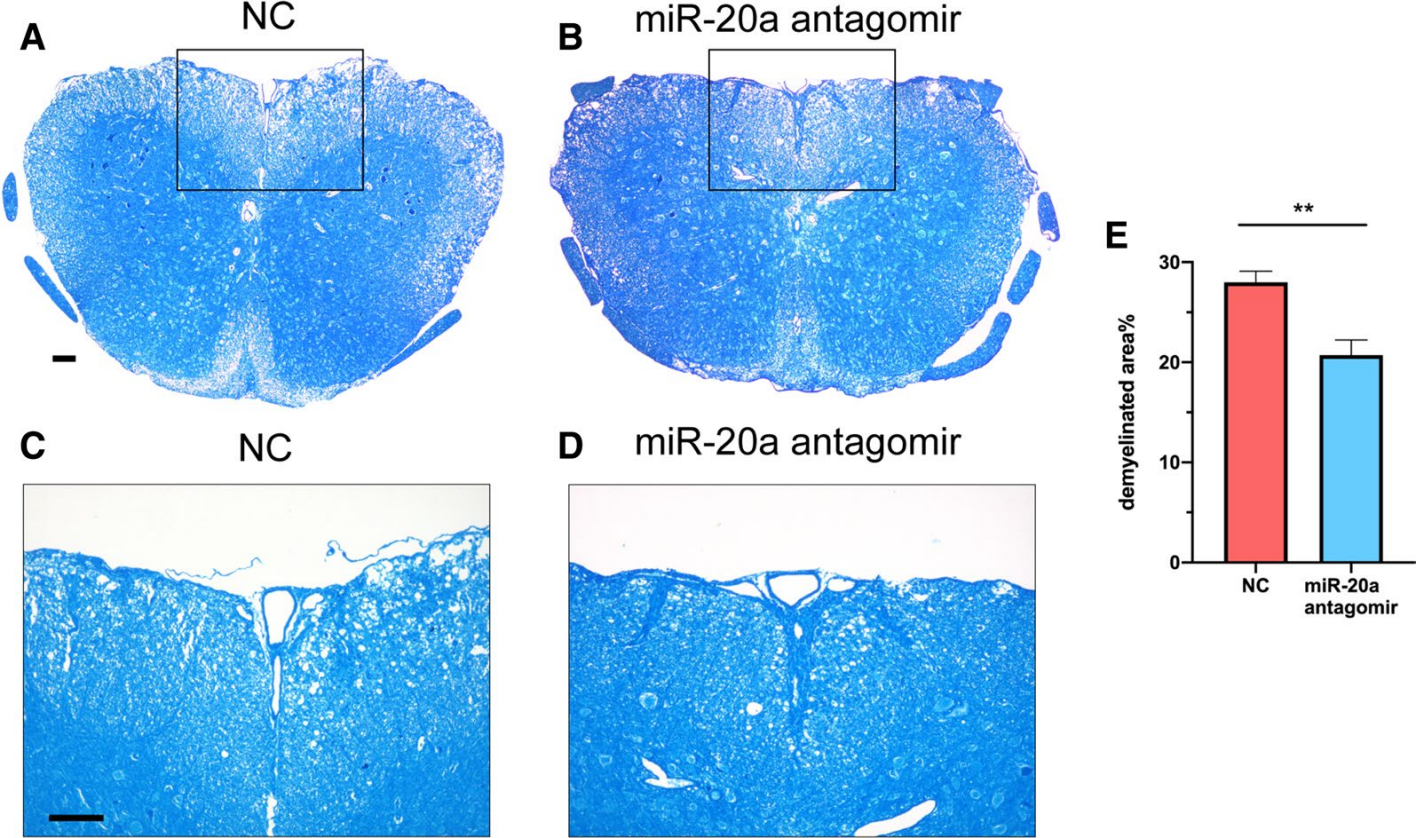
miR-20a antagomir treatment decreases demyelinated areas in spinal cords of EAE mice. **A** Representative Luxol fast blue staining of complete spinal cord sections from NC group (taken at × 10 magnification). **B** Representative Luxol fast blue staining of complete spinal cord sections from miR-20a antagomir group (taken at × 10 magnification). **C** Representative Luxol fast blue staining of partial spinal cord sections from NC group (taken at × 20 magnification). **D** Representative Luxol fast blue staining of partial spinal cord sections from miR-20a antagomir group (taken at × 20 magnification). **E** Quantification of the percentage of demyelinated area to total white matter area in the two groups. Scale bars correspond to 100 μm. The demyelinated areas and total white matter areas were calculated with ImageJ software. Data are shown as mean ± SEM. ** p < 0.01 using unpaired Student’s *t*-test

**Fig. 10:**
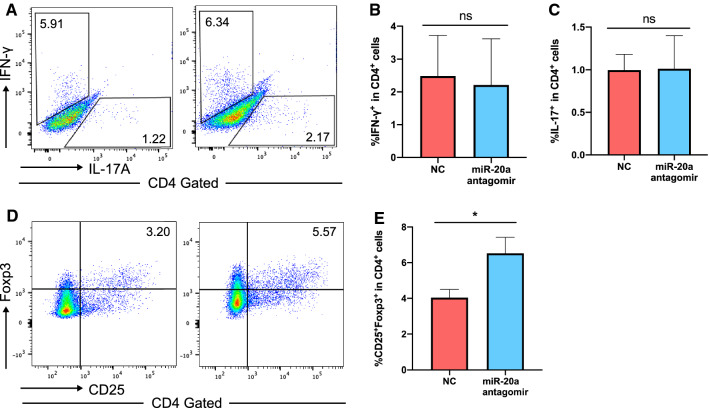
miR-20a antagomir treatment promotes Treg differentiation in EAE mice. **A**–**C** Flow cytometric analysis of IFN-γ^+^ and IL-17A^+^ cells in CD4^+^ T cells of miR-20a antagomir and control group. **D**, **E** Flow cytometric analysis of CD25^+^Foxp3^+^ cells in CD4^+^ T cells of miR-20a antagomir and control group. The flow cytometry plots shown are representative of 4 independent experiments. Data are shown as mean ± SEM. * p < 0.05 using unpaired Student’s *t*-test

We also performed the pathological analysis of spinal cord sections from EAE mice by hematoxylin and eosin (H&E) staining and Luxol fast blue staining. The amount of infiltrating inflammatory cells in the miR-20a antagomir group was less than that in the NC group (Fig. 8A–E). In addition, the percentage of demyelinated area to total white matter area (demyelinated area %) was lower in the miR-20a antagomir group than that in the NC group (Fig. 9A–E). Furthermore, by analyzing the CD4^+^ T cells from the two groups at the remission stage of the disease with flow cytometry, we observed that the percentages of IFN-γ^+^ Th1 cells and IL-17A^+^ Th17 cells in the CD4^+^ gate were similar between the miR-20a antagomir and NC groups (Fig. 10A–C), while the percentage of CD25^+^ Foxp3^+^ Treg cells in the miR-20a antagomir group was higher than that in the NC group (Fig. 10D, E). Collectively, the results showed that miR-20a knockdown with antagomirs alleviates the clinical symptoms of EAE, decreases inflammatory infiltration, reduces myelin damage, and promotes the differentiation of Treg cells in EAE mice. These findings suggest a potential therapeutic role of miR-20a antagomirs in the treatment of EAE and CNS inflammatory demyelinating diseases.

## Discussion

The miR-17-92 cluster was first identified because of its oncogenic effects in a variety of tumors, such as leukemia, lymphoma, and cancers derived from the colon, breast, ovary, and pancreas [22, 24]. In recent years, an increasing number of studies have shown the involvement of miR-17-92 in autoimmune diseases. It is found that miR-17-92 is a critical regulator of follicular helper T (Tfh) cells differentiation in an ovalbumin (OVA)-induced model of autoimmunity [38]. miR-17-92 also plays an essential role in the activation, proliferation, survival, and differentiation of CD4^+^ T cells in graft-versus-host disease (GVHD) [27, 28]. In addition, members of the miR-17–92 cluster were upregulated in CD4^+^ T cells from patients with systemic lupus erythematosus (SLE) [39], and miR-17 affects TNF-α signaling in rheumatoid arthritis [40]. Members of the miR-17-92 cluster are also dysregulated in CNS inflammatory demyelinating diseases, and some of them are restored to normal levels after medical treatment or during remission [30, 32–34, 41–44]. The findings in some studies are contradictory, which may be attributed to the different clinical stages of the patients; however, miR-17-92 cluster is still recognized as an important regulator of CNS inflammatory demyelinating diseases. Nevertheless, the role and mechanism of miR-17-92 cluster in CNS inflammatory demyelinating diseases are still unclear and remain to be further explored.

In this study, we observed the upregulation of miR-20a in splenocytes and lymph node cells from EAE mice and peripheral blood leukocytes from patients with MOG antibody-associated demyelinating diseases and further found that miR-20a was upregulated in CD4^+^ T cells and spinal cord tissues from EAE mice. EAE is mediated mainly by CD4^+^ T cells in which Th1 and Th17 cells are major pathogenic cells while Treg cells play an important role in suppressing inflammatory responses. We speculated that miR-20a may involve in the development of EAE by targeting CD4^+^ T cells. In our study, no evidence showed that miR-20a affects the activation of MOG-specific CD4^+^ T cells, we then found for the first time that miR-20a knockdown promotes the differentiation of MOG-specific CD4^+^ T cells into Tregs, revealing the role of miR-20a in suppressing Treg differentiation in EAE. Jin et al. found that protectin DX increases the proportion of Tregs in a collagen-induced arthritis model by inhibiting NLRP3 inflammasome via miR-20a [45], which appears to be contrary to the findings in our study. However, the animal models used in these two studies are quite different and the important influencing factor in Jin’s study, protectin DX treatment, was not involved in our study, which makes contrasting results in the two studies possible.

In addition, in this study, we observed that miR-20a knockdown using antagomirs in EAE mice in vivo did not affect the proportion of IFN-γ^+^ Th1 and IL-17A^+^ Th17 cells. However, Chang et al. found that miR-20a reduces the proportion of IL-17^+^ cells in CD4^+^ T cells of patients with Vogt-Koyanagi-Harada disease by targeting OSM and CCL1 [46]. We speculate that the differences between their findings and ours are due to the following reasons. First, the diseases investigated in the two studies are different, which leads to completely different pathological states of the CD4^+^ T cells. Second, the results in Chang’s study were obtained through in vitro experiments, whereas our findings were obtained through in vivo experiments, which causes the different results in the two studies.

We further investigated the target gene of miR-20a and demonstrated that *Map3k9* is a functional target gene of miR-20a to suppress Treg differentiation in EAE through luciferase assays and functional validation. Map3k9, also known as mixed-lineage kinase 1 (MLK1), is an important upstream component in the MAPK pathway as a mitogen-activated protein kinase kinase kinase to activate the c-Jun amino-terminal kinase (JNK), p38, and extracellular-signal regulated kinase (ERK) pathways [47–49]. The MAPK signaling pathway plays an important role in a variety of cellular processes, including proliferation, differentiation, survival, apoptosis, and immune response [50]. In previous studies, downstream components of the MAPK signaling pathway have been found to influence multiple activities of Tregs. Bao et al. demonstrated that JNK increases the levels of Foxp3 protein in CD4^+^CD25^+^ Treg cells by binding to its promoters [51]. Lu et al. found that bone morphogenetic protein (BMP)-2/4 promotes the differentiation of Tregs induced by TGF-β through phosphorylated ERK and JNK [52]. In addition, another study showed that inhibiting the activation of p38 abrogates the proliferation and Foxp3 expression in Tregs induced by tumor necrosis factor (TNF) [53]. Furthermore, it has been revealed that the regulatory function of iTregs is associated with enhanced p38 activity [54]. In this study, we found for the first time that an upstream component of the MAPK signaling pathway, Map3k9, enhances the differentiation of Tregs as a direct target of a miRNA, which further extends the findings on the MAPK signaling pathway.

There are still some limitations in this study. First, the optimal dose, timing, and delivery mode of administration of miR-20a antagomirs to treat EAE mice were not investigated in detail. Considering that miR-20a antagomirs are expected to be a treatment for EAE in the future, the issues described above require more intensive exploration. Second, although the results of patients with MOG antibody-associated demyelinating diseases were consistent with those of EAE mice, it is still inconclusive whether the EAE model perfectly simulates MOG antibody-associated demyelinating diseases. In the following studies, we need to isolate CD4^+^ T cells from patients to validate the results of EAE mice. In addition, we did not validate the results of EAE mice in MS patients, which is another limitation. We will validate the results above in MS patients and compare the results of EAE mice, MS patients, and MOG antibody-associated demyelinating diseases patients in the later studies.

In this study, we found for the first time that miR-20a suppresses the Treg differentiation of MOG-specific CD4^+^ T cells in EAE by reducing the expression of Map3k9. In addition, our findings indicate the potential role of miR-20a antagomirs in therapies for EAE and CNS inflammatory demyelinating disease. Since the therapeutic effects of a growing number of miRNAs have been verified in clinical trials [55], we expect miR-20a to play a role in the clinical therapies of CNS inflammatory demyelinating diseases in the future.

## Conclusions

In this study, we demonstrated that miR-20a suppresses the differentiation of MOG-specific CD4^+^ T cells into Tregs in EAE mice by decreasing the expression of Map3k9. Administration of miR-20a antagomirs to EAE mice reduces the severity of the disease, suggesting a potential role of miR-20a in therapies for EAE and CNS inflammatory demyelinating diseases.

## Supplementary Information

**Additional file 1: Table S1.** The primers used in the study.

**Additional file 2: Figure S1.** The knockdown efficiency of the miR-20a antagomirs.

**Additional file 3: Figure S2.** The knockdown efficiency of the Map3k9 siRNA.

## Data Availability

All data are available in the manuscript or upon request to the authors.

## Abbreviations

CNS: Central nervous system
EAE: Experimental autoimmune encephalomyelitis
UTR: Untranslated region
MS: Multiple sclerosis
NMOSD: Neuromyelitis optica spectrum disorder
MOG: Myelin oligodendrocyte glycoprotein
Th: T helper
Treg: Regulatory T cell
iTreg: Induced regulatory T cell
Tfh: Follicular helper T cell
IFN-γ: Interferon-γ
WT: Wild-type
IL: Interleukin
GM-CSF: Granulocyte–macrophage colony-stimulating factor
TGF-β1: Transforming growth factor-β1
Foxp3: Forkhead box P3
RORγt: RAR-related orphan receptor gamma-t
GO: Gene Ontology
KEGG: Kyoto Encyclopedia of Genes and Genomes
Map3k9: Mitogen-activated protein kinase kinase kinase 9
Tgfbr2: Transforming growth factor beta receptor 2
CFA: Complete Freund’s adjuvant
PTX: Pertussis toxin
MACS: Magnetic bead cell sorting
siRNA: Small interfering RNA
NC: Negative control
HEPES: N-2-Hydroxyethylpiperazine-N-2-Ethane Sulfonic Acid
RIPA: Radioimmunoprecipitation assay
EDTA: Ethylenediamine tetraacetic acid
SDS: Sodium dodecyl sulfate
PAGE: Polyacrylamide gel
PVDF: Polyvinylidene fluoride
BSA: Bovine serum albumin
H&E: Hematoxylin and eosin
OVA: Ovalbumin
GVHD: Graft-versus-host disease
SLE: Systemic lupus erythematosus
MLK1: Mixed-lineage kinase 1
JNK: C-Jun amino-terminal kinase
ERK: Extracellular-signal regulated kinase
TNF: Tumor necrosis factor
BMP: Bone morphogenetic protein
