# Supplementary material for: miR-20a suppresses Treg differentiation by targeting Map3k9 in experimental autoimmune encephalomyelitis
Source: J Transl Med. 2021 May 26;19:223. doi: 10.1186/s12967-021-02893-4 (PMC8157414; doi:10.1186/s12967-021-02893-4)
Supplement: Supplementary file 1 — Additional file 1: Table S1. The primers used in the study. [file 12967_2021_2893_MOESM1_ESM.docx]

Additional file 1: Table S1. The primers used in this study

| Gene | Forward primer | Reverse primer |
| --- | --- | --- |
| mmu-miR-17 | CGCAAAGTGCTTACAGTGCAGGTAG | mRQ 3’ Primer (provided in the First-Strand Synthesis Kit) |
| mmu-miR-18a | ACGTAAGGTGCATCTAGTGCAGATAG |  |
| mmu-miR-19a | CGCGTGTGCAAATCTATGCAAAACTGA |  |
| mmu-miR-20a | CGCGTAAAGTGCTTATAGTGCAGGTAG |  |
| mmu-miR-19b | CCGTGTGCAAATCCATGCAAAACTGA |  |
| mmu-miR-92a | TATTGCACTTGTCCCGGCC |  |
| hsa-miR-17 | GCCAAAGTGCTTACAGTGCAGGTAG |  |
| hsa-miR-18a | CGCTAAGGTGCATCTAGTGCAGATAG |  |
| hsa-miR-20a | CGCGTAAAGTGCTTATAGTGCAGGTAG |  |
| hsa-miR-92a | TATTGCACTTGTCCCGGCCTG |  |
| T-bet | AGCAAGGACGGCGAATGTT | GGGTGGACATATAAGCGGTTC |
| RORγt | GACCCACACCTCACAAATTGA | AGTAGGCCACATTACACTGCT |
| Foxp3 | CCCATCCCCAGGAGTCTTG | ACCATGACTAGGGGCACTGTA |
| IL-10 | GCTCTTACTGACTGGCATGAG | CGCAGCTCTAGGAGCATGTG |
| TGF-β1 | CTCCCGTGGCTTCTAGTGC | GCCTTAGTTTGGACAGGATCTG |
| IFN-γ | ATGAACGCTACACACTGCATC | CCATCCTTTTGCCAGTTCCTC |
| IL-17A | TTTAACTCCCTTGGCGCAAAA | CTTTCCCTCCGCATTGACAC |
| IL-17F | TGCTACTGTTGATGTTGGGAC | AATGCCCTGGTTTTGGTTGAA |
| E2f1 | TCACCACAGATCCCAGCCAGTC | TCCTCCCGCACATGCTCCAG |
| Epha4 | GGAAGGAGGGTGGGAGGAAGTG | CCAGTCAGTTCGCAGCCAGTTG |
| Hlf | CCTCCCTACGGCGTGCTCAG | ACGGTCGGGCTGCTGCTC |
| Irak4 | CTTCTCCAGCGACAGCGACAAC | ACCATCCAGGCAGGACAGTCTG |
| Map3k9 | ACGCCAACCAACAGCCTCAAG | CTGCCAGAACAGCTCCACATCC |
| Stat3 | GGGCTTCTCCTTCTGGGTCTGG | CCCGCTCCTTGCTGATGAAACC |
| Tgfbr2 | GGCATCGCTCATCTCCACAGTG | CCCGAAGTCACACAGGCAACAG |
| β-actin | GGCTGTATTCCCCTCCATCG | CCAGTTGGTAACAATGCCATGT |
