# Supplementary material for: miR-20a suppresses Treg differentiation by targeting Map3k9 in experimental autoimmune encephalomyelitis
Source: J Transl Med. 2021 May 26;19:223. doi: 10.1186/s12967-021-02893-4 (PMC8157414; doi:10.1186/s12967-021-02893-4)
Supplement: Supplementary file 2 — Additional file 2: Figure S1. The knockdown efficiency of the miR-20a antagomirs. [file 12967_2021_2893_MOESM2_ESM.docx]

Additional file 2: Figure S1. The knockdown efficiency of the miR-20a antagomirs.


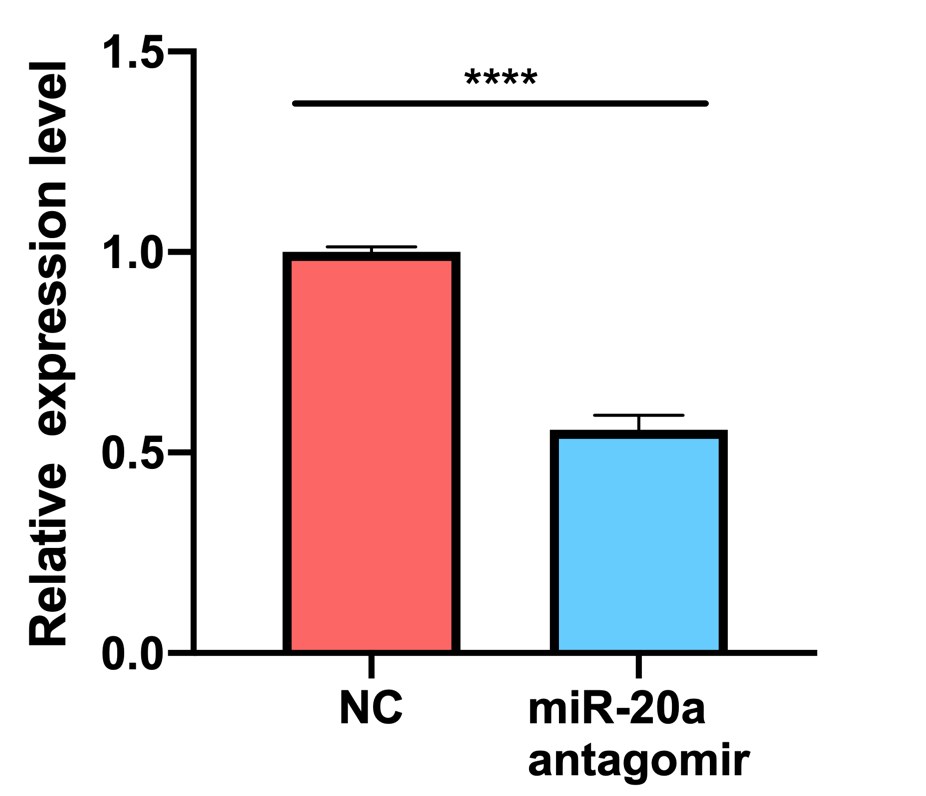


Figure S1. The knockdown efficiency of the miR-20a antagomirs. The expression levels of miR-20a in CD4^+^ T cells transfected with NC or miR-20a antagomirs were detected by qRT-PCR. Data are shown as mean ± SEM. **** p < 0.0001 using unpaired Student’s *t*-test.
