# Supplementary material for: miR-20a suppresses Treg differentiation by targeting Map3k9 in experimental autoimmune encephalomyelitis
Source: J Transl Med. 2021 May 26;19:223. doi: 10.1186/s12967-021-02893-4 (PMC8157414; doi:10.1186/s12967-021-02893-4)
Supplement: Supplementary file 3 — Additional file 3: Figure S2. The knockdown efficiency of the Map3k9 siRNA. [file 12967_2021_2893_MOESM3_ESM.docx]

Additional file 3: Figure S2. The knockdown efficiency of the Map3k9 siRNA.


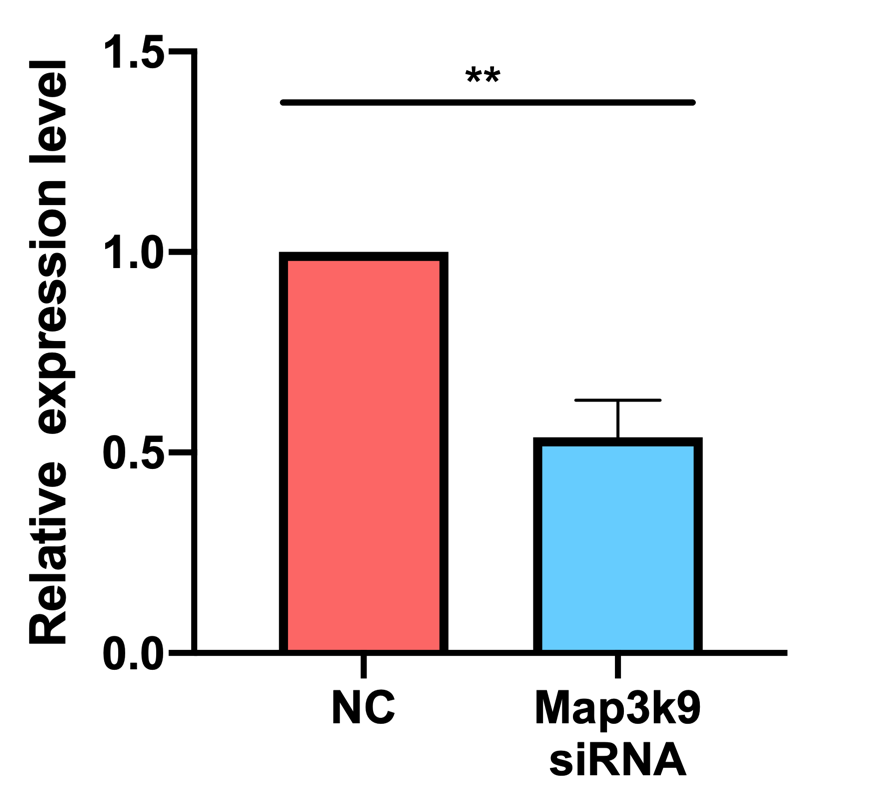


Figure S2. The knockdown efficiency of the Map3k9 siRNA. The expression levels of Map3k9 in CD4^+^ T cells transfected with NC or Map3k9 siRNA were detected by qRT-PCR. Data are shown as mean ± SEM. ** p < 0.01 using unpaired Student’s *t*-test.
